# Adult nasal volumes assessed by acoustic rhinometry

**DOI:** 10.1016/S1808-8694(15)31119-8

**Published:** 2015-10-20

**Authors:** Inge Elly Kiemle Trindade, Adriana de Oliveira Camargo Gomes, Ana Claudia Martins Sampaio-Teixeira, Sergio Henrique Kiemle Trindade

**Affiliations:** 1 Full Professor (Head of the Biological Science Department of the Bauro Dentistry School - USP and Researcher at the Craniofacial Anomalies Rehabilitation Hospital Physiology Laboratory, HRAC-USP, Bauru-SP); 2 Master in Rehabilitation Sciences from the HRAC-USP. (Doctoral student in the Rehabilitation Science post-graduate program, Physiology Laboratory, HRAC-USP, Bauru-SP); 3 Doctor on Sciences from the HRAC-USP (Physiologist, Physiology Laboratory, HRAC-USP, Bauru-SP); 4 Otorhinolaryngologist (Medical doctor in the Otorhinolaryngological Clinical Division at the Clinical Hospital - FMUSP, Servidor Publico Estadual Hospital - SP and Bauru State Hospital - SP)

**Keywords:** nasal cavity, acoustic rhinometry, reference values

## Abstract

**Summary:**

Acoustic rhinometry allows an objective and non-invasive assessment of nasal geometry.

**Aim:**

The present study aimed at determining the volumes of specific segments of the nasal cavity in healthy adults including the nasopharynx, using acoustic rhinometry. Study design: A clinical prospective analysis.

**Cases and Method:**

Thirty volunteers with no evidence of nasal obstruction, aged 18 to 30 years (14 males and 16 females) were analyzed. Volumes were measured at the nasal valve region (V_1_), the turbinates (V_2_), and the nasopharynx (V_3_), before and after application of a topical nasal vasoconstrictor.

**Results:**

The mean volumes measured in 60 cavities before nasal decongestion, were: 1.81±0.35cm^3^ (V_1_), 4.02±1.41cm^3^ (V_2_), and 17.52±4.44cm^3^ (V_3_) for males, and 1.58±0.25cm^3^ (V_1_), 3.94±1.03cm^3^ (V_2_), and 17.80±2.73cm^3^ (V_3_) for females. Gender differences were only significant in V_1_ (p<0.05). After nasal decongestion, the volumes of all the analyzed segments were significantly larger (p<0.05), and the gender differences were significant for V_1_ and V_2_.

**Conclusion:**

Volumes of the three segments in adults with no evidence of nasal obstruction may be used as reference values for other studies.

## INTRODUCTION

Acoustic rhinometry analyzes the cross-sectional area and the volume of the nasal cavity based on reflected sound waves emitted from a source.^3^ The technique is used to check nasal geometry, to identify altered patency, and to monitor the results of surgical procedures on nasal and nasopharyngeal airways.^1^, ^2^, ^3^, ^4^, ^5^, ^6^, ^7^

Hilberg e Pedersen^8^ underlined the importance of creating cross-sectional and volume reference values in their recommendations to the European Rhinological Society on the use of acoustic rhinometry to analyze naso-respiratory function. Nasal volume values based on acoustic rhinometry in normal subjects have been published by various authors using different equipment to analyze nasal segments.^1^, ^2^, ^3^, ^4^,^9^, ^10^, ^11^, ^12^, ^13^, ^14^, ^15^ Chart 1 summarizes the main features raised in published papers and their main findings. It is evident that few studies analyzed more than one nasal cavity segment,^4^,^9^,^11^,^13^ and only one paper^11^ assessed nasopharyngeal volume.

This study aims to define reference values for the volume of three nasal cavity segments in adults with no evidence of nasal obstruction, by using acoustic rhinometry. Our intention is to use this data in other studies of specific populations, such as patients with surgically corrected cleft palate, which are studied at the HRAC-USP physiology laboratory. Additionally, differences between nasal cavities, gender variations, and the effect of nasal vasoconstriction were also analyzed.

## CASES AND METHODS

### Cases

Thirty adult volunteers (14 male and 16 female) aged between 18 and 30 years, with no evidence of nasal obstruction, were studied. All signed a free and informed consent form. Fifty-four volunteers answered a questionnaire based on the Kern model to investigate present and past signs and symptoms of nasal obstruction.^16^ Nasal patency to respiratory flow was measured with a Glatzel mirror placed under the nostrils. Based on this data, 24 subjects with a history of structural nasal anomalies and/or functional disorders, nasal trauma, recurring respiratory infection, regular use of nasal vasoconstrictors, oral breathing, or clearly reduced nasal air flow as shown by the Glatzel mirror (seen only in one patient), were excluded. Thus, with no formal sample size calculation, 30 subjects were included in this study.

The project was approved by the Research Ethics Committee of the Sao Paulo University Craniofacial Anomalies Rehabilitation Hospital, where the study was undertaken.

### Equipment and technique

**Figure 1:**
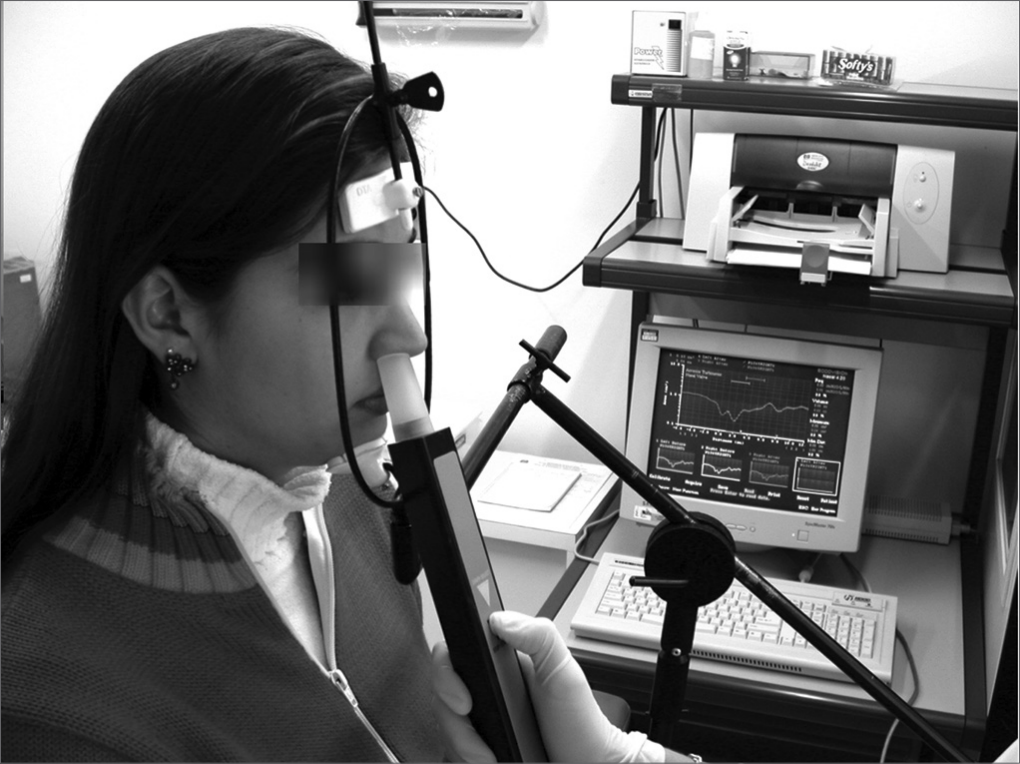
Acoustic rhinometry: equipment used to check nasal volumes.

We used the Eccovision Acoustic Rhinometer (HOOD Laboratories) system for rhinometric assessment. The technique measures reflected sound waves (echoes) in the nose. The equipment includes a source of sound waves (loudspeaker) mounted on the distal end of a 24-cm tube, and a microphone on the proximal end of the tube. The microphone detects, amplifies and digitizes pressure signals, which are fed into a computer running specific software that captures and analyzes the data (figure 1).

**Figure 2:**
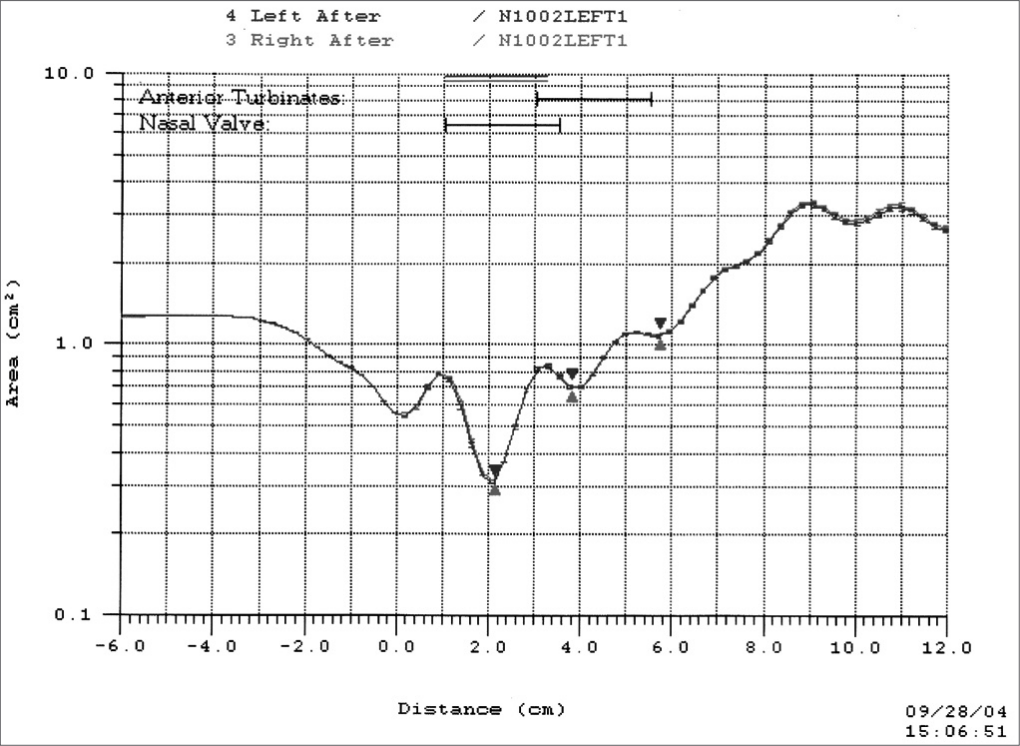
Rhinogram.

The exam proceeds as follows: the proximal end of the rhinometer tube, which is covered with a silicone nosepiece, is placed over one of the nostrils, at a 45-degree angle relative to the nasal floor. Care is taken to avoid deforming the nostril and to create an adequate acoustic seal between the nosepiece and the nostril, with help of a lubricating gel. A sound wave is emitted through the loudspeaker into the nose. Impedance variations caused by constrictions in the cavity under study reflect the sound wave back to the rhinometer tube, into the microphone. The distance from the constriction is calculated from the sound wave velocity and the time it takes for the echo to return. The cross-sectional nasal area is calculated from the echo intensity. Data are converted into an area-distance function which is presented on a chart, which is the rhinogram (figure 2), showing the area (in square centimeters) on the vertical axis as a semi-logarithmic scale, and the distance on the horizontal axis. Volume is calculated from the integration of the area-distance curve. The rhinometer generates 10 sound pulses each 0.5 second and the software calculates the average of the ten repetitions to measure the cross-sectional area and the volume.

### Procedure and variables

Subjects were seated and with their foreheads and chin placed on a support made with orthodontic materials

**Chart 1:** Nasal volumes (V) reported in literature for adults with no evidence of nasal obstruction, before and after nasal vasoconstriction (VC).

Authors	Equipment	n	Age (years)	Race	Condition of patients	Nasal segment	V before VC* (cm^3^)	V after VC* (cm3)
Grymer et al. 1989^1^	Experimental equipment	21	22-48	not specified	no nasal complaints or significant septal deformity	V_0-7cm_		27,15 [1,19]
Grymer et al. 1991^2^	Experimental equipment	82	18-40	not specified	subjective feeling of nasal patency and no evident structural change seen on rhinoscopy	V_0-7cm_	22,60 [0,55]	31,00 [0,57]
Kesavanathan et al. 1995^9^	Experimental equipment	6	20-58	Asians and Caucasians	healthy, non smokers, no history of rhinitis or use of medication that might affect the nasal mucosa	V_0-2,7cm_	3,60 D (1,00)	3,40 (1,20)
							3,30 E (1,10)	3,80 (1,20)
						V_2,2- 7,9cm_	13,90 D (5,50)	13,20 (6,70)
							11,60 E (5,60)	17,40 (5,90)
Morgan et al. 1995^10^	Eccovision Hood Laboratories (AR-1003)	20	33 (10)	Caucasians	no evident structural anomalies, nasal polyps, no past surgery or nasal trauma, recurring upper airway infection, no regular use of nasal medication	V_0-4cm_	4,70 (0,83)	5,59 (0,71)
Roithmann et al. 1995^3^	Eccovision Hood Laboratories (AR-1003)	51 cavities	16-66	not specified	healthy volunteers, with no nasal complaints, no significant functional or structural nasal obstruction	V_0-8cm_	12,14 [0,30]	15,02 [0,30]
Roithmann et al. 1997^4^	Eccovision Hood Laboratories (AR-1003)	66	16-58	not specified	no nasal complaints, no significant functional or structural obstruction or low nasal resistance	V_0-4cm_	3,73	4,23
						V_4-8cm_	7,05	10,18
Tomkinson & Eccles 1998^11^	AR A1 GM Instruments	48	18-59	not specified	no history of nasal diseases; normal anatomy on rhinoscopy	V_0-11cm_	3,44 (0,96)	4,02 (1,18)
						V_11-14cm_	6,99 (2,88)	8,22 (3,15)
						V_14-17cm_	12,56 (5,40)	14,01 (5,82)
Corey et al. 1998^12^	Two microphone AR Hood Laboratories	53	18-57	Caucasians	no obvious nasal deformities, septal deviation, past trauma, nasal surgery, history of allergic rhinitis, nasal polyps, breathing difficulties, use of nasal medication, recent or recurring respiratory infection, or other significant health problems	V_0-6cm_	8,25 (3,23)	11,90 (4,40)
Kunkel et al. 1999^13^	Rhinoklak-1000	15	adultos	not specified	subjective feeling of normal nasal patency	V_0-2,3cm_	4,00 [0,60]	3,60 [0,80]
						V_2,3-4,6cm_	5,70 [1,40]	8,10 [1,60]
						V_4,6-7cm_	8,90 [2,60]	11,10 [2,60]
						V_0-7cm_	18,70 [3,80]	23,40 [4,30]
Silkoff et al. 1999^14^	Eccovision Hood Laboratories (AR-	6	32-48	not specified	no nasal symptoms	V_0-5cm_	5,72 D# (0,27)	
							5,60 E# (0,31)	
Sung et al. 2000^15^	Rhinoklak-RK 1000	20	24,7 (average)	not specified	no septal deviation or rhinopathy	V_0-7cm_	12,98 D (2,27)	
							12,51 E (1,76)	

D: right nasal cavity; E: left nasal cavity

# average values calculated from published individual value

* average (standard deviation) or average [standard error]

specifically for this end (figure 1), to immobilize the head in an axial plane parallel to the ground for rhinometric exams. During data acquisition subjects were asked to hold their breaths at the end of expiration while at rest. Data for three rhinometric curves were collected for each nostril before and 10 minutes after application of 5 drops of a topical nasal vasoconstrictor (xylometazoline chloridrate 0.1%) in each nostril following nasal hygiene, with the subject's head reclined backwards. Rhinograms with irregular tracings or discrepant measurements due to swallowing, head movements or inadequate sealing of the nostrils were discarded. Values considered for analysis were an average of three measurements taken from three technically acceptable curves.

Volume measurements were taken in the following nasal cavity segments: the volume of the segment located from 10 to 32 mm from the nostril corresponding to the nasal valve region (V_1_), the volume of the segment located between 33 and 64 mm from the nostril, corresponding to the turbinate region (V_2_), and the volume of the segment located between 70 and 120mm from the nostril, corresponding to the nasopharyngeal region (V_3_), as recommended in Antilla et al's^6^ study.

### Data analysis

Volume is expressed in cubic centimeters and results for each group are presented as an average ± standard deviation. Student's t test was used to analyze the significance between independent samples (male x female). Student's t test for paired samples was used to analyze the significance of the difference between related samples (right x left nasal cavity, before and after the vasoconstrictor). Values of p<0.05 were considered significant.

## RESULTS

Table 1 shows the average volumes (V_1_, V_2_ and V_3_) of the 30 right nasal cavities (D) and the 30 left nasal cavities (E) of the 30 subjects according to gender, obtained before and after the nasal vasoconstrictor (VC). Statistical analysis revealed no significant difference between right and left measurements in both study groups. Therefore, for simplicity, right and left nasal cavities were considered independent cavities in V_1_ and V_2_. Table 1 also shows the average values of 60 right and left measurements (28 male and 32 female). For V_3_, values obtained from the right and left nasal cavities were not consolidated, as they are measurements of a single cavity obtained from different sides of the nasal cavity. We chose to calculate the right and left average, which resulted in 30 measurements (14 male and 16 female).

**Table 1:** Nasal volumes (V_1_, V_2_ and V_3_) established by acoustic rhinometry in 60 nasal cavities from 30 adults with no evidence of nasal obstruction, according to gender and nasal cavity (right-D and left-E), before and after applying nasal vasoconstriction (VC).

		Before VC			After VC	
Volume (cm^3^)	D	E	D and E	D	E	D and E
male group (n=28) V_1_ (valve)	1,88±0,39 (n=14)	1,74±0,30 (n=14)	1,81±0,35 (n=28)	2,01±0,31 (n=14)	1,82±0,30 (n=14)	1,92±0,32 (n=28)
V_2_ (turbinates)	4,16±1,46 (n=14)	3,89±1,40 (n=14)	4,02±1,41 (n=28)	5,95±0,86 (n=14)	5,80±,89 (n=14)	5,87±0,86 (n=28)
V_3_ (nasopharynx)	17,94±5,30 (n=14)	17,11±5,90 (n=14)	17,52±4,44 (n=14)	23,18±4,32 (n=14)	23,23±4,58 (n=14)	23,21±3,88 (n=14)
female group (n=32) V_1_ (valve)	1,60±0,25 (n=16)	1,55±0,26 (n=16)	1,58±0,25S (n=32)	1,83±0,44 (n=16)	1,72±0,29 (n=16)	1,74±0,27^S^ (n=32)
V_2_ (turbinates)	3,98±1,11 (n=16)	3,89±0,98 (n=16)	3,94±1,03 (n=32)	5,10±1,12 (n=16)	5,36±1,05 (n=16)	5,23±1,08^S^ (n=32)
V_3_ (nasopharaynx)	17,82±3,36 (n=16)	17,79±4,03 (n=16)	17,80±2,73 (n=16)	22,30±4,17 (n=16)	22,23±4,84 (n=16)	22,27±4,24 (n=16)

average ± standard deviation

n= number of nasal cavities analyzed

S p<0.05 statistically significant difference (male vs. female for D and E

We found that the average right and left nasal volume (V_1_ and V_2_) in the female group was lowe than the corresponding volume in the male group, before and after using the vasoconstrictor. However, statistical analysis demonstrated that the differences weer significant only for V_1_ values before and after vasoconstrictor use, and significant for V_2_ volumes only after vasoconstrictor use.

The nasopharyngeal volume (V_3_) did not differ significantly between males and females.

Male and female data were consolidated to study the effect of vasoconstriction on nasal volume. Averages for the whole group and percentage variations due to the vasoconstrictor are presented on Table 2. Statistical analysis revealed that values obtained after the vasoconstricto were significantly higher compared to pre-vasoconstriction volumes in all three nasal segments. V_2_ (39%) and V_3_ (24%) variations were more pronounced than V1 (08%) variations.

**Table 2:** Nasal volumes (V_1_, V_2_ and V_3_) established by acoustic rhinometry in 60 nasal cavities from 30 male and female adults with no evidence of nasal obstruction, according to gender and nasal cavity (right-D and left-E), before and after applying nasal vasoconstriction (VC).

Volume (cm^3^)	Before VC	After VC	Percentage variation
V_1_ (valve)	1,68±0,32 (n=60)	1,82±0,30 S (n=60)	8%
V_2_ (turbinates)	3,98±1,21 (n=60)	5,53±1,03 S (n=60)	39%
V_3_ (nasopharynx)	17,67±3,57 (n=30)	22,72±4,06 S (n=30)	29%

average ± standard deviation

n = number of nasal cavities analyzed

S p<0.05: statistically significant difference (before vs. after VC)

## DISCUSSION

This study aimed to define reference volumes for specific segments of the nasal cavity in adults. As mentioned above, initially 54 apparently healthy subjects were selected, from which a sample of 30 subjects with a subjective feeling of normal nasal patency were chosen, a criterion also adopted by other authors.^5^,^9^,^14^,^19^, ^20^, ^21^ These subjects, according to their answers in a questionnaire, did not have a history of nasal alterations. The questionnaire assessment was considered adequate for sample selection, as a preliminary analysis of 24 subjects not included in the study - due to evidence of nasal disorders - showed that the volume and area of segments V1 and V2 were significantly lower in this group than in those groups with no complaints. We were also able to find that measurement variability, expressed by the standard deviation, was comparable to that reported by Corey et al^12^ for normal subjects, which have a more homogeneous profile than individuals with variable degrees of nasal obstruction.

We also underline that only young adults with no evident African or Asian physical traits were selected; nasal cavity dimensions depend not only on age but also on race.^8^,^10^,^12^ Also, our analysis of 30 patients in fact corresponded to measurements from 60 nasal cavities, which is a significant number for the aims of this study.

Our critical analysis is based on Hilberg and Pedersen's8 recommendations. There are many causes of errors in measuring the internal nasal dimensions using acoustic rhinometry, as shown by various authors.^8^,^17^, ^18^, ^19^,^22^ These include ambient temperature variations and external noise that can reduce the accuracy and reproducibility of the technique. These variables were not controlled for in our study; however, measurements were done always in the same room, which had a relatively stable temperature and a maximum noise level of 60dB. Before each exam, subjects answered the questionnaire and were instructed about the procedure; this stage took about 30 minutes, enough time for adaptation to the environment.

Other causes of errors according to Hilberg and Pedersen^8^ are changes in the position of the rhinometer and sound loss due to maladjustment of the nasal adaptor and the nostril. We were careful to uniformly place the tube always parallel to the dorsum of the nose. Neutral electrocardiogram gel was used between the nasal adaptor and the nostril to assure adequate sealing, as recommended in literature. Due care was also taken to avoid deforming the nostril and consequently, the nasal valve. The Eccovision Acoustic Rhinometer has an adaptor that only touches the nostril and is not introduced into the nasal vestibule (as was the case with older olive-shaped models), which in itself avoids deformation of the nasal valve. Spectacles were also removed to avoid external pressure on the nose.

Additional care was taken to maintain the head in a stable position during rhinometry. A special frame was developed to support the chin and the forehead so that measurements could be done with the head in a stable position and parallel to the ground. These last recommendations do not appear in Hilberg and Pedersen8 paper, however, other studies^18^,^22^ have stated that postural changes interfere on measurements, and suggest controlling the head position, with which we agree. Similarly, breathing and swallowing were also mentioned as factors that may interfere with rhinogram measurements and quality.^18^,^22^ Subject were thus asked to close their mouths, to hold their breaths, and not to swallow or move their tongue during data acquisition (which takes only a few seconds).

On the whole these strategies were used to increase the consistency of results and to reduce as much as possible those factors that could interfere on the measurements. Additionally, calibration of the equipment was done at the beginning of each period for each day, curves with irregularities were discarded, and those values used for analysis were calculated from the average of three technically acceptable curves. Having used these procedures in the past in our laboratory, we were able to obtain variation coefficients between 6% and 8% in our rhinometric volume measurements.^23^

With this background, we analyze and compare our results with published papers. This comparison is somewhat limited due to the fact that many authors who assessed nasal volumes in subjects with no evidence of nasal obstruction^1^, ^2^, ^3^, ^4^,^6^,^9^, ^10^, ^11^, ^12^,^14^,^15^ studied different segments from those used by Antila et al,^6^ on which our study is based, or else reported bilateral measurements.^13^ On the other hand, Antila et al^6^ analyzed patients with evidence of nasopharyngeal obstruction, complaints of snoring and sleep apnea, which also compromises a comparison. Nevertheless, Antila et al6 obtained similar average results for V_1_ (2.03±0.48 for the right side, and 2.04±0.53 for the left side) and V_2_ (3.49±1.20 for the right side, and 3.40±1.05 for the left side), corroborating our findings. The average value for V_3_ (9.47±3.13 for the right side, and 9.11±3.34 for the left side) was markedly lower, which may be explained by the diseases the subjects presented.

The utility of measurements in those segments we assessed has to be discussed; V_1_ corresponds to the nasal valve region (1.0 to 3.2cm from the nostril), V_2_ is the region of the turbinates (3.3 to 6.4cm), and V_3_ is the nasopharynx (7 to 12cm). As seen on Chart 1, points and segments chosen for analysis are not uniform in published papers. Some authors combined segments and others chose different regions. We believe that segmentation of measurements into nasal valve region, turbinate region and nasopharynx, as proposed by Antila,6 is the preferred option to meet the aims of our study.

There was a tendency for women to have lower volumes than men, different from what is stated in Grymer et al's2 paper, who reported the opposite situation for volumes calculated between 0-7cm before nasal vasoconstriction. Although we found a statistical significance for these measurement differences only in anterior nasal cavity regions (V_1_ before and V_1_ and V_2_ after nasal vasoconstriction) we suggest using male and female nasal volumes separately, as reference values. This does not apply to the nasopharynx, where there was no gender difference.

Nasal vasoconstriction aims to identify structural changes in the nasal fossae by abolishing the functional effect produced by the nasal mucosa. As expected, data analysis revealed that values obtained after nasal vasoconstriction were significantly higher. The decongestion effect was more evident in V2, meaning that the region of the turbinates reacted most to vasoconstriction, confirming past results.^2^, ^3^, ^4^,^10^, ^11^, ^12^

Finally, we emphasize that data on nasopharyngeal volumes should be analyzed with care, as other studies have shown that systematic or random errors may be introduced in measurements done in the posterior region of the nasal cavity. This may be due, for instance, to marked anterior vasoconstriction resulting in underestimated values, or sound reflection to the contralateral cavity or the paranasal sinuses, or changes in the tonus of the pharyngeal muscles and involuntary movement of the soft palate during the exam.^3^,^24^, ^25^, ^26^, ^27^, ^28^ Furthermore, a recent study on normal subjects^29^ compared measurements done by acoustic rhinometry and computed tomography, which is considered a gold standard, and showed a good correlation (r=0.839) between both methods for assessments made up to an average distance of 6cm from the nostrils. Beyond this point, the correlation is reduced (r=0.419), showing that acoustic rhinometry systematically underestimates true measurements. Based on these findings, we concluded that acoustic rhinometry, compared to computed tomography, provides accurate measurements up to the turbinates, with lower accuracy in posterior regions. Evidence also shows that these technical limitations do not invalidate the clinical usefulness of this method for posterior regions of the nasal cavity. The method may be employed in comparisons in the same subject, such as when investigating relative volume variations caused by velar movement in silent speech,^30^, ^31^, ^32^ or to analyze variations caused by surgery (tonsillectomies,^33^,^34^ septoplasty/turbinectomy^1^,^35^,^36^, or maxillomandubilar osteotomy^37^,^38^), taking into account that systematic errors are common, that random errors may be minimized, and that measurements are reproducible in the same subject.^29^

## CONCLUSION

The different volumes verified in this study are representative of internal nasal dimensions in adults with no nasal obstruction, and may be taken as reference values for comparative studies involving populations with various nasal diseases.
